# Submucosal Partial Denture in the Esophagus: A Case of Foreign Body Removal

**DOI:** 10.7759/cureus.90899

**Published:** 2025-08-24

**Authors:** Christopher Park, John DeWitt, Michael W Sim

**Affiliations:** 1 Otolaryngology - Head and Neck Surgery, Indiana University Health, Indianapolis, USA; 2 Gastroenterology, Indiana University Health, Indianapolis, USA; 3 Otolaryngology - Head and Neck Surgery, Indiana University School of Medicine, Indianapolis, USA

**Keywords:** dental appliance, endoscopic, esophagus, foreign body, surgery

## Abstract

A male in his 30s presented to a gastroenterology clinic and subsequently to an otolaryngology - head and neck surgery clinic for a one-month history of progressive dysphagia. X-ray was unrevealing, and CT demonstrated non-specific esophageal wall thickening inferior to the post-cricoid area. Esophagoscopy by head and neck surgery in the operating room revealed a submucosal protrusion of the posterior wall of the cervical esophagus. After unroofing the overlying mucosa, a broad, flat lesion was grasped, removed, and identified as a large plastic device consistent with a partial denture. The patient did not experience any post-operative complications, and a follow-up esophagram on post-operative day seven showed no evidence of leak. Manual manipulation techniques, including biopsy attempts, can play an important role in unroofing an apparent submucosal foreign body in the esophagus.

## Introduction

While the majority of esophageal foreign bodies pass without complication, it is estimated that 1500 deaths occur annually as an esophageal foreign body-related complication [1,2]. Though the exact incidence of foreign body ingestion is unknown, higher incidences are seen in the pediatric population, patients with cognitive impairments, and patients with underlying esophageal disease, including motility disorders and luminal pathologies [1]. While much of the data on esophageal foreign bodies is retrospective, some studies purport that dental appliances are one of the leading causes of esophageal foreign body (FB) impaction after food bolus and bones [1,3,4]. In most instances, patients present with the chief complaint of dysphagia or chest pain, with imaging revealing a radio-opaque FB within the esophagus [3,5]. While small, topographically simple FBs are often removed via traditional rigid esophagoscopy, larger and more topographically complex objects may require other types of scope equipment or even an open thoracic or cervical approach, due to increased risk of esophageal perforation, particularly with sharp or longer objects [6,7]. A systematic review by Aiolfi et al. demonstrated that the most frequent site of impaction was the cervical esophagus, most commonly caused by sharp objects, with retrosternal pain being the most common presenting symptom, followed by dysphagia. This review also noted that of the 13,092 cases examined, a surgical approach to disimpaction was required in 3.4% of patients, and the overall mortality rate was 0.85% [2].

## Case presentation

Patient history

A male in his 30s was referred to a gastroenterology (GI) clinic for a one-month history of progressive dysphagia, initially to small pills progressing to solid food. Notably, the patient had a history of gastroesophageal reflux disease (GERD) on pantoprazole daily, was a current smoker (minimal; five cigarettes per day for two years), and did not drink alcohol. He reported a 20 lb weight loss over the last month due to a gradual inability to intake solids and relying exclusively on protein shakes and liquids for nutrition. He also reported a tightness on the right side of his neck that developed after making constant swallow attempts while eating. He states that he had been taking pantoprazole chronically and consistently, without return of his regular symptoms, including heartburn and mild hoarseness in the morning. He denied any known ingestion of foreign bodies. Patient denied difficulty breathing, dysphonia, emesis, hematemesis, or hemoptysis. He initially presented to gastroenterology and thoracic surgery (history and workup below) but eventually presented to the emergency room due to unremitting dysphagia. The patient had no prior head or neck surgeries and no family history of head and neck or esophageal cancers or other esophageal diseases.

Investigations

**Figure 1:**
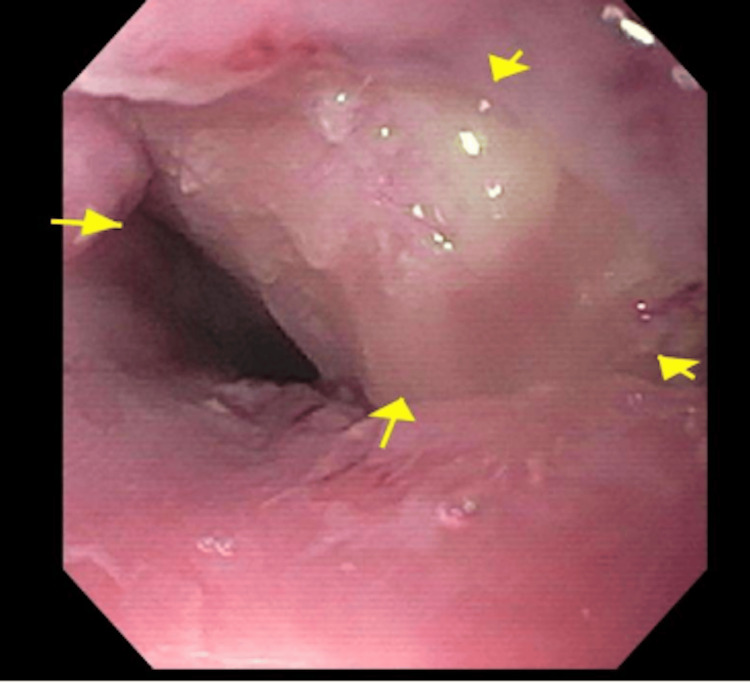
Endoscopic view of the proximal esophagus with an apparent extrinsic obstructive mass (arrows).

Two months prior to presentation to the emergency room, the patient underwent esophagogastroduodenoscopy (EGD), which revealed what appeared to be a stenosis of the cervical esophagus and no other significant findings. Dilation was performed at that time up to 9 mm using a Savary dilator (Bloomington, IN: Cook Medical). Resistance was noted at the level of the cricopharyngeus at that time. The patient had mild improvement of dysphagia initially, but developed worsening symptoms within a week of treatment. Therefore, repeat EGD was performed two weeks later, showing inflammation of the cervical esophagus with an apparent submucosal mass measuring 1.8 cm. The overlying mucosa was smooth but more pale than normal. The X-ray was unrevealing. A CT scan showed partial circumferential cervical esophageal wall thickening inferior to the post-cricoid region with a corresponding cystic abnormality. An esophagram again demonstrated narrowing of the cervical esophagus without obstruction. A third EGD was performed six weeks later, which showed cervical esophageal wall thickening measuring approximately 2 x 1.5 cm, but no other findings or evidence of FB (Figure 1). The esophagus was dilated to 27 Fr at that time, and a fine needle aspiration (FNA) was performed, which was non-diagnostic. A tissue biopsy was also attempted; however, this resulted in the structural failure of the endoscopic biopsy forceps.

At this point, the patient was referred to head and neck surgery for further evaluation, but presented to the emergency room prior to the scheduled appointment due to worsening oral (PO) intake with liquids and 15 lb weight loss. The physical examination was unremarkable, with no neck tenderness, no decreased range of motion in the neck, and no palpable neck masses. Flexible laryngoscopy was unremarkable.

Differential diagnosis

Given the inconclusive imaging findings, the differential diagnosis at this time was broad, including a foreign body, esophageal stricture, and esophageal mass (both benign and malignant). A malignant mass was less likely given the patient’s age, acute onset of symptoms, minimal tobacco use, and alcohol use history, and well-controlled GERD history. Esophageal stricture was also less likely given these same factors, as well as the patient’s lack of head and neck surgical history. Thus, an esophageal foreign body was the leading differential.

Treatment

**Figure 2:**
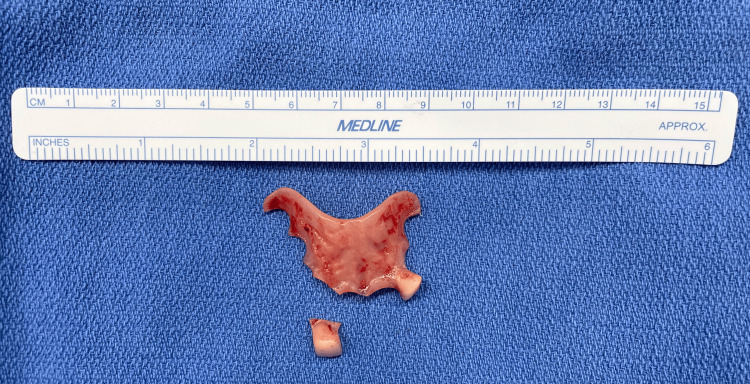
Partial denture and detached tooth.

**Figure 3:**
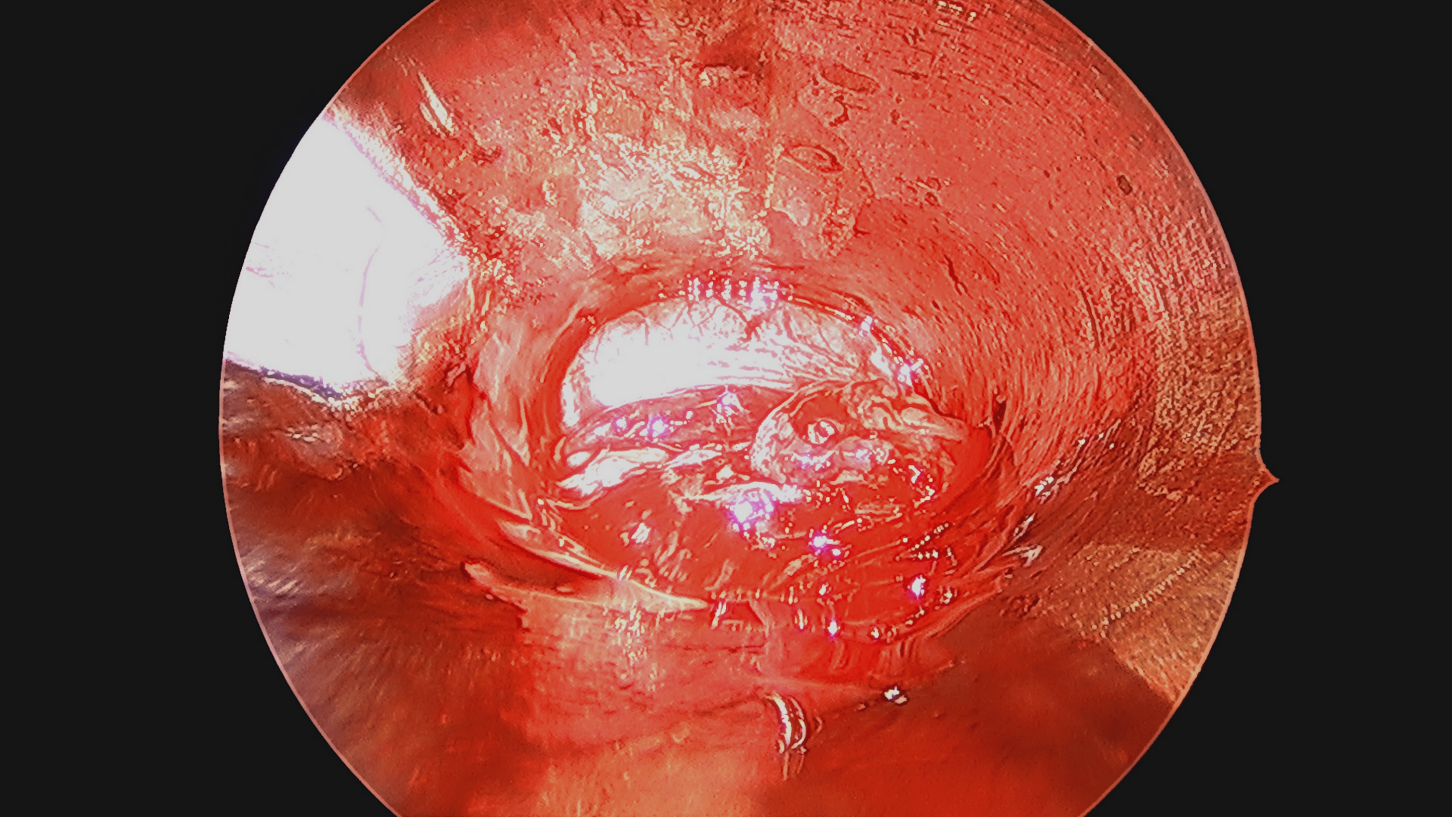
Endoscopic view of the proximal esophagus after removal of the submucosal foreign body.

The patient was taken to the operating room for direct laryngoscopy and rigid esophagoscopy by head and neck surgery. Findings included a submucosal protrusion of the posterior wall of the cervical esophagus with subsequent occlusion of the esophageal lumen, impeding passage of the rigid scope beyond this point. A biopsy was attempted by unroofing the overlying mucosa, revealing a firm, white lesion that appeared to be dentition. The lesion was grasped with alligator forceps and removed, which was identified to be a synthetic tooth (Figure 2). A second look revealed a broad, flat lesion, which was grasped, removed, and identified as a large plastic device consistent with a partial denture (Figure 2). Final endoscopic examination showed no additional foreign bodies of the esophagus, patent esophageal lumen, and 50% transverse mucosal tear of the posterior wall from which the objects were removed (Figure 3). Histopathological examination revealed fragments of ulcer with acute and chronic inflammation, accompanied by scant reactive squamous cells.

Outcome and follow-up

The patient did not have any post-operative complications. The patient was admitted for two nights of observation, kept nothing by mouth (NPO) for one week, and a follow-up esophagram on post-operative day seven showed no evidence of a leak. The patient noted continued improvement with no new symptoms or issues. Patient had a follow-up EGD with the GI team several weeks later, and no evidence of stenosis was found. The patient has been able to swallow normally since the operation.

## Discussion

This case report describes a unique case of mucosalized FB in the esophagus. While the majority of FBs in the esophagus pass spontaneously without complication, identification of a retained FB is especially critical due to the mechanical risk of perforation or fistulization, as well as other complications, such as pneumonia, mediastinitis, and esophageal stricture [8,9]. While several case reports of dental and oral appliance FB removals have been published, none to our knowledge describe an instance of a patient presenting with chronic dysphagia that had serial diagnostic workups without clear evidence of FB [3,9,10]. Furthermore, this patient did not have any known history of foreign body ingestion or known predisposing factors. In this scenario, one proposed mechanism of injury would be blunt force injury to the esophageal wall and lodging of the denture with eventual submucosal tracking of the foreign body over time due to compressive forces from subsequent food boluses. We were not able to ascertain which layers were initially injured due to the friable appearance of the tissue upon removal.

Esophageal FB typically presents with retrosternal pain or dysphagia with or without other associated symptoms, such as hoarseness, difficulty breathing, and chest pain [11]. Other cases, however, may be asymptomatic [9]. A thorough history should be obtained with particular focus on the use of oral appliances, day and night-time use if present, smoking history, family history of head and neck cancer, weight loss, and the aforementioned symptoms. The initial workup for cervical dysphagia should also include a flexible laryngoscopy [12]. A negative laryngoscopy should prompt further evaluation via imaging and possible esophagoscopy [6,12]. While X-ray imaging is diagnostic for radiopaque objects, it is unable to identify radiolucent FBs [12]. Contrast studies may also be normal or demonstrate findings inconsistent with the classical FB appearance, such as a “cystic mass,” as in this case [13].

Esophagoscopies are the traditional gold standard for esophageal FB assessment and removal [5]. However, as illustrated in this case, direct visualization may only reveal normal or ulcerative mucosa. More specifically, investigations may reveal the entire FB, partial FB, ulceration without FB, or even the appearance of normal mucosa with extrinsic compression, depending on the time at which endoscopy is undertaken and progression of FB impaction and mucosalization. In the current case, an attempt at biopsy of an apparent submucosal mass unroofed the overlying mucosa, revealing the FB and allowing for successful removal.

Importantly, this case illustrates that even in rigorous and repeated evaluations, a foreign body can elude identification and that a mucosalized, radiolucent FB can uniquely masquerade as a submucosal mass. Therefore, thorough evaluations, including attempts to biopsy such lesions, can prove to be a valuable approach.

## Conclusions

This case illustrates the importance of direct visualization with careful, tactile examination and possible biopsy of the mucosal and submucosal space of the esophagus when there is an apparent extrinsic or submucosal mass, especially when accompanied by a history of chronic dysphagia, suspicion for FB, and/or multiple prior procedures. Moreover, while the majority of dental appliances are radiopaque, many modern appliances are radiolucent and may evade diagnostic imaging. Thus, in the setting of ongoing dysphagia with apparent extrinsic compression of the esophagus, a mucosalized FB must be considered.
